# Effect of preadmission glucocorticoid therapy on 30-day mortality in critically ill patients: a retrospective study of a mixed ICU population in a tertiary hospital

**DOI:** 10.1186/s13613-019-0489-8

**Published:** 2019-01-18

**Authors:** Tak Kyu Oh, In-Ae Song, Jae Ho Lee, Cheong Lim, Young-Tae Jeon, Hee-Joon Bae, You Hwan Jo

**Affiliations:** 1 Department of Anesthesiology and Pain Medicine, Seoul National University Bundang Hospital, Gumi-ro 173 Beon-gil, Bundang-gu, Seongnam, Korea; 2 Division of Pulmonary and Critical Care Medicine, Department of Internal Medicine, Seoul National University Bundang Hospital, Gumi-ro 173 Beon-gil, Bundang-gu, Seongnam, Korea; 3 Department of Thoracic and Cardiovascular Surgery, Seoul National University Bundang Hospital, Gumi-ro 173 Beon-gil, Bundang-gu, Seongnam, Korea; 4 Department of Neurology, Stroke Center, Seoul National University Bundang Hospital, Gumi-ro 173 Beon-gil, Bundang-gu, Seongnam, Korea; 5 Department of Emergency Medicine, Seoul National University Bundang Hospital, Gumi-ro 173 Beon-gil, Bundang-gu, Seongnam, Korea

**Keywords:** Glucocorticoid, Mortality, Intensive care unit

## Abstract

**Background:**

This study aimed to investigate the association between preadmission glucocorticoid (GC) therapy and 30-day mortality in critically ill patients following admission to an intensive care unit (ICU). We aimed to determine whether this association differed according to daily GC dosage and type. We conducted a retrospective cohort study of adult patients admitted to a single tertiary academic hospital ICU from January 2012 to December 2017. We classified the patients regularly undergoing oral GC therapy as preadmission GC users, and those with no history of GC use were classified as non-GC users.

**Results:**

The study included 24,929 patients, of whom 816 (3.3%) were preadmission GC users. Thirty-day mortality in preadmission GC users (173 of 816 patients) was 21.2% compared to 8.8% (2113 of 24,113 patients) in non-GC users. Multivariate Cox regression analysis showed that preadmission GC users had a 1.62-fold increase in 30-day mortality compared to non-GC users [hazard ratio (HR) 1.62, 95% confidence interval (CI) 1.29–2.03, *P *< 0.001]. When comparing preadmission GC users with diabetes mellitus to non-GC users, a 2.29-fold increase in 30-day mortality was noted (HR 2.29, 95% CI 1.08–4.86, *P *= 0.031). In the sensitivity analysis, compared to non-GC users, daily dosages of ≤ 5 and > 5 mg of prednisolone in preadmission GC users showed 1.45-fold (HR 1.45, 95% CI 1.03–2.03, *P *= 0.033) and 1.67-fold (HR 1.67, 95% CI 1.25–2.24, *P *= 0.001) increases, respectively, in 30-day mortality after ICU admission. Moreover, prednisolone, methylprednisolone, and dexamethasone users in the preadmission GC users group showed 1.56-fold (HR 1.56, 95% CI 1.21–2.01, *P *= 0.001), 1.90-fold (HR 1.90, 95% CI 1.12–3.25, *P *= 0.018), and 1.30-fold (HR 1.30, 95% CI 1.05–1.50, *P *= 0.042) increases, respectively, in 30-day mortality compared to non-GC users.

**Conclusion:**

Preadmission GC use among critically ill patients was associated with an increased 30-day mortality after ICU admission compared to non-GC use. This association was more prevalent in preadmission GC users with diabetes mellitus and in preadmission GC users who took > 5 mg/day of prednisolone and methylprednisolone.

**Electronic supplementary material:**

The online version of this article (10.1186/s13613-019-0489-8) contains supplementary material, which is available to authorized users.

## Background

The steroid hormone glucocorticoid (GC) is a pleiotropic hormone that is commonly prescribed for 1.2% [1] and 1.0% [2] of patients with chronic conditions in the USA and in the UK, respectively. GC has both immunosuppressive and potent anti-inflammatory effects [3], and GC inhibits the immune response and the production of prostaglandins and leukotrienes, the two main products of inflammation [4]. GC has been reported to be a useful treatment option worldwide for patients who suffer from diseases such as allergies [5], chronic obstructive lung disease or asthma [6], autoimmune disease [7], and rheumatic disease [8], due to its immunosuppressive and anti-inflammatory effects. However, chronic GC use is known to cause various side effects [9], of which patient susceptibility to infection due to its immunosuppressive effects is the most fatal [10]. Moreover, chronic GC use has a direct immunosuppressive effect that leads to an increased risk of infection [10], and chronic GC use can also lead to central adrenal failure [11]. In this regard, chronic GC use prior to admission to an intensive care unit (ICU) could increase the mortality risk, especially in critically ill patients more susceptible to infection and mortality due to sepsis [12]. Many patients admitted to the ICU experience newly ICU-acquired immunosuppression for various reasons [13]; therefore, preadmission GC use that can reduce immunity in patients may be associated with a poor prognosis in critically ill patients. One cohort study has reported that preadmission GC use increased 30-day mortality after stroke [14], and another cohort study showed a decrease in the incidence of acute respiratory distress syndrome (ARDS) in sepsis patients [15]. However, few studies have investigated the effects of preadmission GC use in critically ill patients; therefore, this issue remains challenging.

This study aimed to investigate the association between preadmission GC use and 30-day mortality after ICU admission among adult patients. Additionally, we aimed to determine whether this association differed according to daily dosage, types of GC, or the main diagnosis at the time of ICU admission.

## Materials and methods

### Study design and participants

This retrospective cohort study investigated the medical records of adult patients aged > 18 years who had been admitted to an ICU from January 2012 to December 2017. If a patient had been admitted to the ICU more than once during the study period, only details of the last ICU admission were included for analysis. Patients with incomplete or missing medical records detailing medication history were excluded from the analysis. All data collection and classifications were performed by a medical records technician from the institution who was blinded to the study purpose.

### Ethics

This study was conducted with the approval of the Seoul National University Bundang Hospital Institutional Review Board (IRB) (IRB approval number: B-1806/474-105). The requirement for informed consent was waived by the IRB as this was a retrospective study.

### Preadmission chronic GC use (independent variable)

Patients were classified as preadmission GC users if they had been taking a daily dose of oral GC prior to ICU admission. Conversely, patients were defined as non-GC users if they had no history of GC use. Oral GCs were classified into three types, namely prednisolone, methylprednisolone, and dexamethasone, and daily GC dosages were divided into two groups, namely > 5 mg/day and ≤ 5 mg/day of prednisolone. Using this dosage calculation, 4 mg/day of methylprednisolone and 0.75 mg/day of dexamethasone were considered the same as a dosage of 5 mg/day of prednisolone, given the potency of GCs [16]. Our reasoning for dividing the groups according to a 5 mg/day cutoff was that previous studies have reported no significant association with doses < 5 mg prednisolone for all-cause mortality in patients with rheumatoid arthritis [17, 18].

### Covariates

Data collected for this study included demographic information such as sex, age (in years), body mass index (kg/m^2^), and medication history at ICU admission, and the Acute Physiology and Chronic Health Evaluation II score at ICU admission. Comorbidity data at ICU admission concerning hypertension, diabetes mellitus, ischemic heart disease, cerebrovascular disease, chronic obstructive lung disease, liver disease (liver cirrhosis, hepatitis, and fatty liver disease), chronic kidney disease, anemia (hemoglobin < 10 g/dl), and cancer were collected. Information concerning whether admission occurred through the emergency department was also obtained. Additionally, the main diagnosis on ICU admission, for example, septic shock, cardiac disease, neurologic disease, kidney failure, respiratory insufficiency or failure, drug intoxication, trauma, gastrointestinal bleeding/ischemia/or perforation, hemorrhagic shock, post-cardiac arrest, and others [liver failure, urinary tract infection, peripheral vascular disease, and ICU admission for close monitoring], was also recorded. The main diagnosis on ICU admission was classified using ICU records completed by the primary physician at the time of ICU admission. If, on ICU admission, one patient had multiple main diagnoses, for example, septic shock with kidney failure, then the patient was assigned to two groups (septic shock and kidney failure) in duplicate.

### Outcome measure (dependent variable)

Thirty-day mortality was defined as any death that occurred within 30 days of ICU admission. The accurate death date for all patients, including patients who had been discharged, was obtained up until May 16, 2018, with approval from the Korean Ministry of the Interior and Safety.

### End points

The primary end point was to identify the association between preadmission GC therapy and 30-day mortality after ICU admission. Thirty-day mortality due to diagnosed infection was confirmed in cases where the pathogen had been confirmed through culture. Additionally, we investigated whether this association changed according to dosage, types of GC, and main diagnosis on ICU admission.

### Statistical analysis

To compare the clinical and demographic characteristics based on preadmission GC use, sample t-tests for continuous variables and Chi-square tests for categorical variables were used. First, a univariate Cox regression analysis was performed to identify the individual correlation between the covariates and 30-day mortality. From this univariate analysis, covariates with a *P* value < 0.2 were selected for adjustment in the final multivariate Cox regression analysis.

Additionally, sensitivity analyses according to daily dosage and type of GC were performed to investigate whether the association between chronic GC use and 30-day mortality might differ according to daily dosage and type of GC taken by the patients in the chronic GC user group. Finally, a subgroup analysis of 11 groups, according to the main diagnosis at the time of ICU admission, was performed to confirm whether an association between chronic GC use and 30-day mortality might differ according to the main diagnosis at the time of ICU admission. In this subgroup analysis, Bonferroni correction was applied to reduce type I errors in multiple comparisons [19]. We tested the interaction of chronic GC use and a function of time, implemented as time-dependent chronic GC use, to confirm that the proportional hazards assumption had been satisfied (*P* > 0.05). All statistical analyses were performed using IBM SPSS version 24.0. (IBM Corp., Armonk, NY). A *P*-value < 0.005 was considered statistically significant for the subgroup analysis using Bonferroni correction, and *P* < 0.05 was considered statistically significant for all other analyses.

## Results

**Fig. 1:**
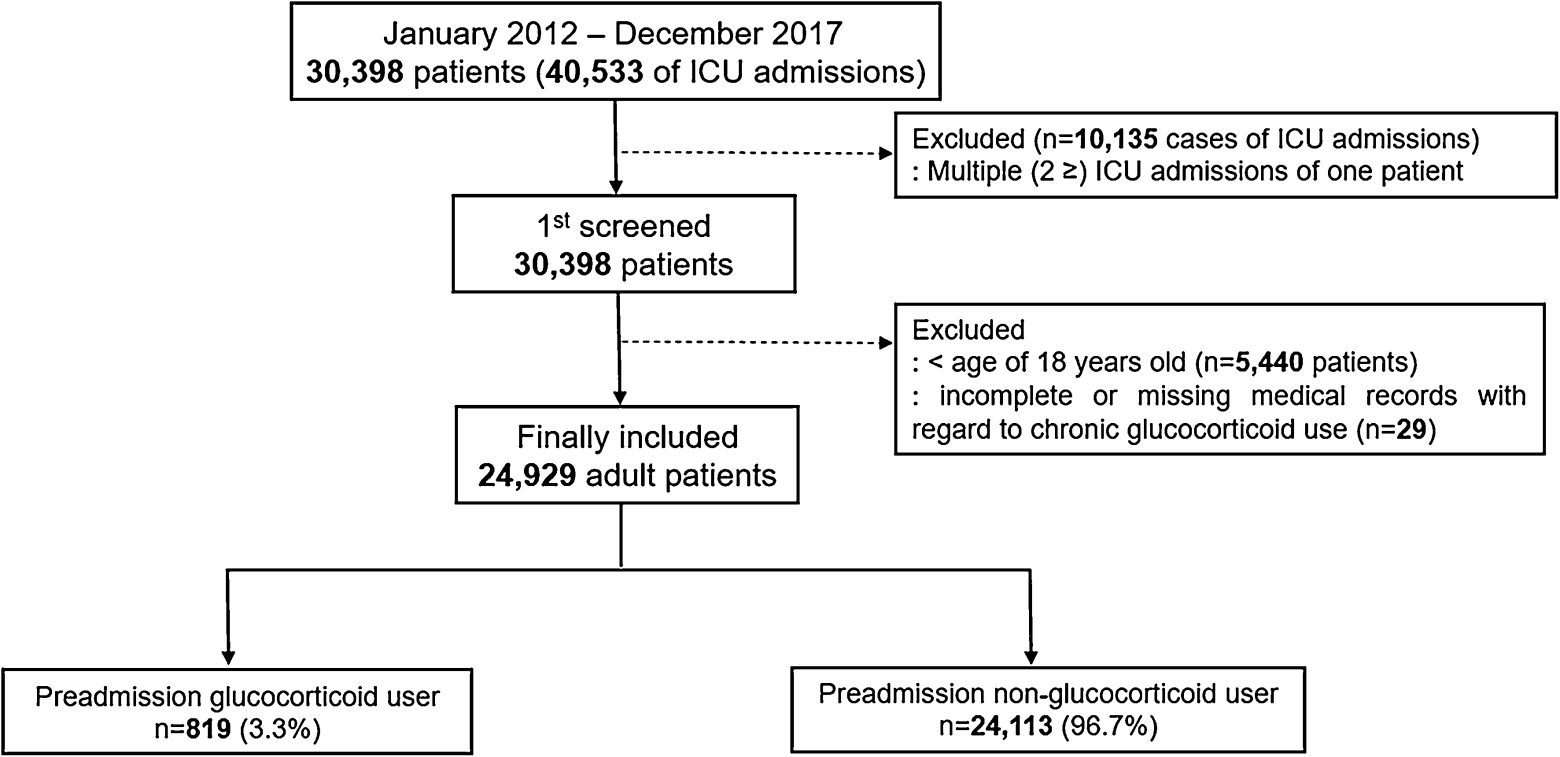
Flowchart describing patient selection. *ICU* intensive care unit

From January 2012 to December 2017, a total of 30,398 patients had been admitted to the ICU on 40,533 occasions. Of them, 10,135 ICU admissions were excluded because they represented multiple admissions, and only the last episode of ICU admission was considered for each patient. Next, 5440 pediatric patients < 18 years of age, and 29 patients with incomplete or missing medical records with regard to chronic GC use, were excluded. In total, 24,929 patients were included in the analysis, and of them, 819 (3.3%) patients were preadmission GC users (Fig. 1). The 30-day mortality was 21.2% (173 of 816 patients) in the preadmission GC users group and 8.8% (2113 of 24,113 patients) in the non-GC users. Thirty-day mortality due to diagnosed infection occurred in 31 of 816 (3.8%) preadmission GC users and in 260 of 24,113 (1.1%) non-GC users.

### Thirty-day mortality after ICU admission

Table 1 shows the differences in baseline characteristics between preadmission GC users and non-GC users, and 30-day mortality was significantly higher in preadmission GC users (173 of 816 patients, 21.2%) than in non-GC users (2113 of 24,113 patients, 8.8%) (*P *< 0.001). Additional file 1: Table S1 shows the univariate Cox regression analysis of the association between covariates and 30-day mortality after ICU admission. Table 2 shows the results of the Cox regression analysis for the association between 30-day mortality and preadmission GC use before and after adjustment for the covariates selected from the univariate analysis. In the adjusted multivariate Cox regression model, there was a 1.62-fold increase in 30-day mortality when comparing preadmission GC users and non-GC users [hazard ratio (HR) 1.62, 95% confidence interval (CI); 1.29–2.03, *P *< 0.001]. Additionally, there was a 2.29-fold increase in the 30-day mortality when preadmission GC users with diabetes mellitus and non-GC users were compared (HR 2.29, 95% CI 1.08–4.86, *P *= 0.031).

**Table 1:** Comparison of characteristics between preadmission glucocorticoid users and preadmission non-glucocorticoid users

Variables	Preadmission GC group (*n* = 816)	Preadmission non-GC group (*n* = 24,113)	*P*-value
Sex: male	442 (54.2%)	14,434 (59.9%)	0.001
Age (years)	65.2 (14.8)	63.4 (15.6)	0.001
Body mass index (kg/m^2^)	22.9 (4.0)	23.8 (3.8)	< 0.001
APACHE II	20.9 (10.5)	18.9 (10.2)	< 0.001
Comorbidities at ICU admission			
Hypertension	335 (41.1%)	10,559 (43.8%)	0.121
Diabetes mellitus	80 (9.8%)	2275 (9.4%)	0.723
Ischemic heart disease	26 (3.2%)	661 (2.7%)	0.445
Cerebrovascular disease	37 (4.5%)	1057 (4.4%)	0.836
Chronic obstructive lung disease	59 (7.2%)	993 (4.1%)	< 0.001
Liver disease (LC, hepatitis, fatty liver)	42 (5.1%)	709 (2.9%)	< 0.001
Chronic kidney disease	152 (18.6%)	3749 (15.5%)	0.017
Anemia (Hb < 10 g/dl)	358 (43.9%)	7348 (30.5%)	< 0.001
Cancer	214 (26.2%)	4147 (17.2%)	< 0.001
Admission through the emergency department	487 (59.7%)	12,122 (50.3%)	< 0.001
Postoperative ICU admission	287 (35.2%)	10,384 (43.1%)	< 0.001
Main diagnosis at ICU admission			
Septic shock	56 (6.9%)	353 (1.5%)	< 0.001
Cardiac disease	190 (23.3%)	8002 (33.2%)	< 0.001
Neurologic disease	111 (13.6%)	3237 (13.4%)	0.883
Kidney failure	72 (8.8%)	1653 (6.9%)	0.029
Respiratory insufficiency or failure	211 (25.9%)	2695 (11.2%)	< 0.001
Drug intoxication	6 (0.7%)	170 (0.7%)	0.919
Trauma	6 (0.7%)	185 (0.8%)	0.918
GI bleeding, ischemia, or perforation	34 (4.2%)	1074 (4.5%)	0.695
Hemorrhagic shock	2 (0.2%)	105 (0.4%)	0.413
Post-cardiac arrest	19 (2.3%)	312 (1.3%)	0.011
Others*	286 (35.0%)	9236 (38.3%)	0.327
Length of hospital stay (days)	16.4 (36.8)	11.1 (18.7)	< 0.001
Length of ICU stay (days)	2.6 (9.3)	5.3 (26.7)	< 0.001
All-cause 30-day mortality	173 (21.2%)	2113 (8.8%)	< 0.001
30-Day mortality due to diagnosed infection	31 (3.8%)	260 (1.1%)	< 0.001

Presented as number (percentage) or mean value (standard deviation)

*T* test for continuous variables and Chi-square test for categorical variables were used

*APACHE* Acute Physiology and Chronic Health Evaluation, *GC* glucocorticoid, *GI* gastrointestinal, *Hb* hemoglobin, *ICU* intensive care unit, *LC* liver cirrhosis

Others* include liver failure, urinary tract infection, unknown, peripheral vascular disease, and ICU admission for monitoring

**Table 2:** Cox regression analysis for 30-day mortality after ICU admission according to preadmission GC use

Variable	30-day mortality	
	Hazard ratio (95% CI)	*P*-value
Unadjusted model		
Preadmission non-GC user	1	
Preadmission GC user	2.78 (2.38, 3.24)	< 0.001
Covariates adjusted model*		
Preadmission non-GC user	1	
Preadmission GC user	1.62 (1.29, 2.03)	< 0.001
Preadmission GC user with diabetes mellitus	2.29 (1.08, 4.86)	0.031

*APACHE* Acute Physiology and Chronic Health Evaluation, *CI* confidence interval, *GC* glucocorticoid, *GI* gastrointestinal, *ICU* intensive care unit

*Covariates of *P *< 0.2 in univariate Cox regression analysis in Additional file 1: Table S1 were included in multivariate Cox regression analysis: age, sex, body mass index, APACHE II, comorbidity at ICU admission (hypertension, diabetes mellitus, diabetes mellitus with chronic GC use, cerebrovascular disease, chronic obstructive lung disease, liver disease, chronic kidney disease, anemia, and cancer), admission through emergency department, postoperative ICU admission, main diagnosis on ICU admission (septic shock, cardiac disease, kidney failure, respiratory insufficiency or failure, trauma, GI bleeding, ischemia or perforation, and post-cardiac arrest)

### Sensitivity analysis according to dosage and types of GCs

The results of sensitivity analysis according to dosage and types of GCs are presented in Table 3. Compared to non-GC users, daily dosages of ≤ 5 mg and > 5 mg in preadmission GC users showed 1.45-fold (HR 1.45, 95% CI 1.03–2.03, *P *= 0.033) and 1.67-fold (HR 1.67, 95% CI 1.25–2.24, *P *= 0.001) increases in 30-day mortality after ICU admission. Additionally, prednisolone users, methylprednisolone users, and dexamethasone users from within the preadmission GC users group showed 1.56-fold (HR 1.56, 95% CI 1.21–2.01, *P *= 0.001), 1.90-fold (HR 1.90, 95% CI 1.12–3.25, *P *= 0.018), and 1.30-fold (HR 1.30, 95% CI 1.05–1.50, *P *= 0.042) increases in 30-day mortality after ICU admission compared to non-GC users.

**Table 3:** Sensitivity analysis according to types and daily dosage of glucocorticoids

Variable	30-Day mortality	
	Hazard ratio (95% CI)	*P*-value
Daily dosage*		
Preadmission non-GC user (*n* = 24,113)	1	(0.001)
Preadmission GC user of daily dosage ≤ 5 mg (*n* = 393)	1.45 (1.03, 2.03)	0.033
Preadmission GC user of daily dosage > 5 mg (*n* = 423)	1.67 (1.25, 2.24)	0.001
Type of GCs*		
Preadmission non-GC user (*n* = 24,113)	1	(0.001)
Preadmission GC user: prednisolone (*n* = 635)	1.56 (1.21, 2.01)	0.001
Preadmission GC user: methylprednisolone (*n* = 131)	1.90 (1.12, 3.25)	0.018
Preadmission GC user: dexamethasone (*n* = 50)	1.30 (1.05, 1.50)	0.042

*APACHE* Acute Physiology and Chronic Health Evaluation, *CI* confidence interval, *GC* glucocorticoid, *GI* gastrointestinal, *ICU* intensive care unit

*Covariates of *P *< 0.2 in univariate Cox regression analysis in Additional file 1: Table S1 were included in multivariate Cox regression analysis: age, body mass index, APACHE II, comorbidity at ICU admission (hypertension, cerebrovascular disease, liver disease, chronic kidney disease, anemia, and cancer), admission through emergency department, main diagnosis on ICU admission (septic shock, cardiac disease, neurologic disease, kidney failure, respiratory insufficiency or failure, GI bleeding, ischemia or perforation, and post-cardiac arrest)

### Subgroup analysis according to the main diagnosis on ICU admission

The results of subgroup analysis according to the main diagnosis at the time of ICU admission are presented in Table 4. Thirty-day mortality was 40.1% (164 of 409 patients) for septic shock, 4.2% (340 of 8192) for cardiac disease, 4.7% (156 of 3348) for neurologic disease, 27.1% (468 of 1725) for kidney failure, 25.5% (740 of 2906) for respiratory insufficiency or failure, 6.2% (11 of 176) for drug intoxication, 7.9% (15 of 191) for trauma, 8.7% (96 of 1108) for gastrointestinal bleeding/ischemia/or perforation, 6.5% (7 of 107) for hemorrhagic shock, and 54.4% (180 of 331) for post-cardiac arrest.

**Table 4:** Subgroup analysis according to the main diagnosis at the time of ICU admission

Variable	30-Day mortality	
	Hazard ratio (95% CI)	*P*-value*
Septic shock (*n* = 409)		
Preadmission GC user	3.21 (1.72, 5.97)	< 0.001
Cardiac disease (*n* = 8192)		
Preadmission GC user	1.49 (0.65, 3.43)	0.345
Neurologic disease (*n* = 3348)		
Preadmission GC user	1.90 (1.67, 5.40)	< 0.001
Kidney failure (*n* = 1725)		
Preadmission GC user	1.07 (0.53, 2.16)	0.860
Respiratory insufficiency or failure (*n* = 2906)		
Preadmission GC user	2.04 (1.47, 2.82)	< 0.001
Drug intoxication (*n* = 176)		
Preadmission GC user	0.00 (0.00–)	0.966
Trauma (*n* = 191)		
Preadmission GC user	0.00 (0.00–)	0.984
GI bleeding, ischemia, or perforation (*n* = 1108)		
Preadmission GC user	0.55 (0.12, 2.59)	0.453
Hemorrhagic shock (*n* = 107)		
Preadmission GC user	0.00 (0.00–)	1.00
Post-cardiac arrest (*n* = 331)		
Preadmission GC user	2.33 (0.87, 6.25)	0.093
Others** (*n* = 9522)		
Preadmission GC user	1.83 (0.52, 3.25)	0.138

Covariates of *P *< 0.2 in univariate Cox regression analysis in Additional file 1: Table S1 (except the main diagnosis of ICU admission) were included in multivariate Cox regression analysis: age, body mass index, APACHE II, comorbidity at ICU admission (hypertension, cerebrovascular disease, liver disease, chronic kidney disease, anemia, and cancer), admission through emergency department

*APACHE* Acute Physiology and Chronic Health Evaluation, *CI* confidence interval, *GC* glucocorticoid, *GI* gastrointestinal, *ICU* intensive care unit

*P < 0.005 was considered statistically significant after Bonferroni correction

Others**include liver failure, urinary tract infection, unknown, peripheral vascular disease, and ICU admission for monitoring

In multivariate Cox regression analysis, preadmission GC users showed 3.21-fold (HR 3.21, 95% CI 1.72–5.97, *P *< 0.001), 1.90-fold (HR 1.90, 95% CI 1.67–5.40), and 2.04-fold (HR 2.04, 95% CI 1.47–2.82, *P *< 0.001) increases in 30-day mortality after ICU admission in septic shock patients, neurologic disease, and respiratory insufficiency or failure patients compared to non-GC users, respectively.

## Discussion

This study showed that preadmission GC use was significantly associated with an increase in 30-day mortality among critically ill adult patients. This association was more prevalent in preadmission GC users with diabetes mellitus and in preadmission GC users who took a higher daily dosage, such as > 5 mg/day of prednisolone. Additionally, in the subgroup analysis, preadmission GC users who had been diagnosed with septic shock or respiratory insufficiency or failure, and neurologic disease on ICU admission showed an associated increase in 30-day mortality after ICU admission. This study is the first to investigate the association between preadmission GC use and 30-day mortality in a mixed ICU population.

First, our findings reflect those of a previous study that showed a direct immunosuppressive effect due to preadmission chronic GC use might be associated with 30-day mortality in critically ill patients [10]. This possible association is supported by the results of our subgroup analysis indicating that critically ill patients with septic shock or respiratory failure or insufficiency were more affected if they had been chronic GC users. Septic shock or respiratory insufficiency might be due to infection such as pneumonia [20], and the immunosuppressive effect of chronic GC use on 30-day mortality could be significant in these patients. Second, adrenal insufficiency, commonly due to chronic GC administration [21], might affect 30-day mortality in critically ill patients. A recently published systematic review has reported that the median incidence of adrenal insufficiency among chronic GC users was 37.4% (interquartile range 13–63%) [22]. Given that mortality has been reported to increase in patients with adrenal insufficiency [23], adrenal insufficiency due to chronic GC use may have affected the results in this study.

Previous studies have not shown consistent results regarding the effects of chronic GC use on the mortality of critically ill patients. Sundbøll et al. [14] reported that preadmission GC use was associated with an increase in 30-day mortality among patients with ischemic stroke, intracranial hemorrhage, and subarachnoid hemorrhage, and their results are similar to the findings in our study that showed 30-day mortality increased in patients with neurologic disease on ICU admission (Table 4). However, McKown et al. [15] reported that there was no significant association between preadmission GC use and hospital mortality among sepsis patients, although it was associated with a lower incidence of early acute respiratory distress syndrome (ARDS). The contrasting conclusions in McKown et al.’s study and in our study may be due to differing population characteristics. In McKown et al.’s study, patients admitted to ICU with sepsis were included for analysis, whereas we included a mixed ICU population for analysis; therefore, the effects of preadmission GC use may have differed.

The results of sensitivity analysis according to daily dosage were also noteworthy in this study. Two previous studies showed that a low daily GC dose (≤ 5 mg of prednisolone) did not affect mortality in patients with rheumatoid arthritis [17, 18]. However, a daily GC dose of ≤ 5 mg of prednisolone was associated with an increase in 30-day mortality in critically ill patients in our study. There are two possible explanations for this. First, critically ill patients are more severely ill than are patients with rheumatoid arthritis, and the immunosuppressive effect of chronic GC administration might be a significant risk factor for mortality in those taking a low daily dose of GC (≤ 5 mg of prednisolone). Second, the systematic review reported that adrenal insufficiency could have occurred in patients receiving a daily dose of < 5 mg prednisolone, which might have affected 30-day mortality among critically ill patients in that study [22].

Preadmission GC users with diabetes mellitus showed a relatively higher risk of 30-day mortality after ICU admission than other preadmission GC users compared to non-GC users. Chronic GC administration is known to be a risk factor for the development of diabetes mellitus [24]. Moreover, GC administration usually causes hyperglycemia [25], and management of hyperglycemia in chronic GC users is a challenging issue facing clinicians. A previous study reported that hyperglycemia in hospitalized patients who received GC administration was common, and it was associated with higher mortality or morbidity in the patients [26]. Therefore, the findings in our study suggest that preadmission GC users with diabetes mellitus on ICU admission should be treated even more carefully because these patients have an increased risk of 30-day mortality.

This study has several limitations. First, due to the study’s retrospective cohort design, selection bias may have been present. Second, this cohort study was conducted at a single center; therefore, the generalizability of our findings may be limited. Third, having to perform a dose conversion for the different GCs to conduct a sensitivity analysis according to daily dosage when considering the potency of each GC could have introduced bias into this study. Finally, since this study used records from multiple physicians determining the main diagnosis on ICU admission, there may have been inconsistencies or inaccuracy in determining the diagnoses.

In conclusion, the present study showed that preadmission GC use among critically ill patients was associated with increased 30-day mortality after ICU admission compared to non-GC use. This association was more prevalent in preadmission GC users with diabetes mellitus and preadmission GC users taking > 5 mg/day of prednisolone or methylprednisolone. Additionally, this association was significant in patients who had been admitted for septic shock, respiratory insufficiency or failure, and neurologic disease.

## Additional file

**Additional file 1.** **Table S1.** Univariate Cox regression analysis of covariates for 30-day mortality after ICU admission.

## Abbreviations

APACHE: Acute Physiology and Chronic Health Evaluation
ARDS: acute respiratory distress syndrome
CI: confidence interval
GC: glucocorticoid
GI: gastrointestinal
HPA: hypothalamic pituitary adrenal
HR: hazard ratio
ICU: intensive care unit
IRB: institutional review board
