# Supplementary material for: Effect of preadmission glucocorticoid therapy on 30-day mortality in critically ill patients: a retrospective study of a mixed ICU population in a tertiary hospital
Source: Ann Intensive Care. 2019 Jan 18;9:8. doi: 10.1186/s13613-019-0489-8 (PMC6338611; doi:10.1186/s13613-019-0489-8)
Supplement: Supplementary file 1 — Additional file 1. Table S1. Univariate Cox regression analysis of covariates for 30-day mortality after ICU admission. [file 13613_2019_489_MOESM1_ESM.docx]

Table S1. Univariate Cox regression analysis of covariates for 30-day mortality after ICU admission

| Variables | | Hazard Ratio (95% CI) | *P*-value |
| --- | --- | --- | --- |
| Sex: male | | 1.10 (1.01, 1.20) | 0.023 |
| Age, year | | 1.04 (1.03, 1.04) | <0.001 |
| Body mass index, kg m^-2^ | | 0.89 (0.87, 0.90) | <0.001 |
| APACHE II | | 1.06 (1.06, 1.06) | <0.001 |
| Comorbidities at ICU admission | |  |  |
|  | Hypertension | 0.92 (0.85, 1.00) | 0.043 |
|  | Diabetes mellitus | 1.41 (1.24, 1.61) | <0.001 |
|  | Diabetes mellitus with chronic GC use | 3.52 (2.15, 5.76) | <0.001 |
|  | Ischemic heart disease | 0.97 (0.72, 1.30) | 0.821 |
|  | Cerebrovascular disease | 1.30 (1.08, 1.58) | 0.007 |
|  | Chronic obstructive lung disease | 1.23 (1.03, 1.47) | 0.026 |
|  | Liver disease (LC, hepatitis, fatty liver) | 2.63 (2.24, 3.08) | <0.001 |
|  | Chronic kidney disease | 3.70 (3.41, 4.03) | <0.001 |
|  | Anemia (Hb <10 g dl^-1^) | 4.02 (3.69, 4.37) | <0.001 |
|  | Cancer | 2.53 (2.31, 2.76) | <0.001 |
| Admission through the emergency department | | 5.23 (4.67, 5.86) | <0.001 |
| Postoperative ICU admission | | 0.29 (0.26, 0.33) | <0.001 |
| Main diagnosis at ICU admission | |  |  |
|  | Septic shock | 5.96 (5.08, 6.99) | <0.001 |
|  | Cardiac disease | 0.51 (0.45, 0.57) | <0.001 |
|  | Neurologic disease | 0.90 (0.76, 1.06) | 0.215 |
|  | Kidney failure | 4.23 (3.82, 4.68) | <0.001 |
|  | Respiratory insufficiency or failure | 4.67 (4.27, 5.10) | <0.001 |
|  | Drug intoxication | 1.19 (0.66, 2.16) | 0.564 |
|  | Trauma | 1.47 (0.88, 2.44) | 0.141 |
|  | GI bleeding, ischemia or perforation | 1.79 (1.46, 2.20) | <0.001 |
|  | Hemorrhagic shock | 1.37 (0.65, 2.89) | 0.401 |
|  | Post-cardiac arrest | 12.23 (10.47, 14.27) | <0.001 |
|  | Others* | 1.24 (0.52, 1.95) | 0.324 |

All covariates of *P* < 0.2 in univariate Cox regression analysis were included in the multivariate Cox regression analysis.

Others* include liver failure, urinary tract infection, unknown, peripheral vascular disease, and ICU admission for monitoring.

APACHE, acute physiology and chronic health evaluation; GC, glucocorticoid; Hb, Hemoglobin; ICU, intensive care unit; LC, liver cirrhosis
